# Ectopic mediastinal parathyroid diagnosed by contrast-enhanced endoscopic ultrasound-guided fine-needle aspiration and eluate parathormone level

**DOI:** 10.1055/a-2516-2740

**Published:** 2025-02-06

**Authors:** Matteo Parolin, Antonino Caruso, Paola Sartorato, Andrea Lisotti, Stefano Benvenuti, Ernesto De Menis, Pietro Fusaroli

**Affiliations:** 1 18703 Medicina Generale 2, Ospedale Santa Maria di Ca Foncello, Treviso, Italy; 2 18703 Gastroenterology, Ospedale Santa Maria di Ca Foncello, Treviso, Italy; 3 9296 Gastroenterology and Digestive Endoscopy Unit, Hospital of Imola, Università di Bologna, Imola, Italy

Primary hyperparathyroidism is defined as an elevated serum calcium with an elevated or inappropriately normal parathyroid hormone (PTH), and rarely it can be due to an ectopic mediastinal parathyroid 1 .

We describe a 68-year-old woman with a new diagnosis of primary hyperparathyroidism due to a suspected mediastinal parathyroid. Laboratory tests revealed an elevated serum calcium (11.5 mg/dL [normal range 8.8–10.1]), decreased serum phosphorus (2.1 mg/dL [2.5–4.5]), high parathormone (150 pg/mL [12–72]), and hypercalciuria (313 mg/day [>250 mg/day]). The patient also had multiple co-morbidities, including atrial fibrillation, obesity, and a multinodular goiter. According to recent guidelines, parathyroidectomy was indicated 1 .

We performed endoscopic ultrasound (EUS) to identify the precise location of the ectopic parathyroid, using a linear echoendoscope (GF-UCT180; Olympus Medical, Tokyo, Japan) connected to an Arietta S70 (Hitachi-Aloka, Japan) 2 3 . We detected an ill-defined hypoechoic inhomogeneous lesion (19 × 8 mm) at 20 cm from the upper incisors on the right side of the esophagus ( Video 1 ). As we deemed B-mode scanning insufficient to proceed with EUS-guided fine-needle aspiration (EUS-FNA) safely, we investigated the lesion further with contrast-enhanced EUS after injection of one vial of Sonovue (Bracco, Italy), which showed a hyperenhanced lesion 4 . EUS-FNA was then performed with a 22G needle (Expect; Boston Scientific, USA). The aspirate was diluted in 2 mL normal saline and a real-time rapid PTH assay was performed. The cytologic appearance and PTH level on the diluted eluate (>2500 pg/mL) were diagnostic of an ectopic parathyroid ( Fig. 1 ).

Sequential steps of endoscopic ultrasound-guided fine-needle aspiration (EUS-FNA) of an ectopic mediastinal parathyroid.Video 1

For the detection of ectopic adenomas, current guidelines 1 recommend imaging methods such as neck ultrasound, scintigraphy, positron-emission tomography, and computed tomography, which may be inconclusive in some cases. EUS-FNA and PTH testing on the eluate, which is usually more sensitive than the cytology of the specimen, should be considered. We believe that EUS-FNA is safe in these often co-morbid and frail patients, allowing appropriate referral for a major surgical intervention such as parathyroidectomy 5 .

**Fig. 1:**
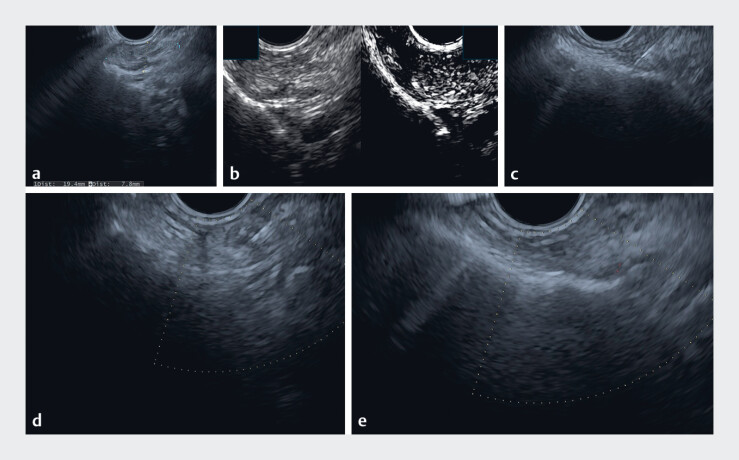
Endoscopic ultrasound (EUS) images showing the stages of the EUS-guided fine-needle aspiration (EUS-FNA) including: **a** identification of the parathyroid; **b** the use of contrast-enhanced EUS; **c, d** the EUS-FNA procedure being performed; **e** the appearance after the EUS-FNA.

Endoscopy_UCTN_Code_CCL_1AF_2AC

## Notes

Endoscopy E-Videos https://eref.thieme.de/e-videos
*E-Videos* is an open access online section of the journal *Endoscopy* , reporting on interesting cases and new techniques in gastroenterological endoscopy. All papers include a high-quality video and are published with a Creative Commons CC-BY license. Endoscopy E-Videos qualify for HINARI discounts and waivers and eligibility is automatically checked during the submission process. We grant 100% waivers to articles whose corresponding authors are based in Group A countries and 50% waivers to those who are based in Group B countries as classified by Research4Life (see: https://www.research4life.org/access/eligibility/ ). This section has its own submission website at https://mc.manuscriptcentral.com/e-videos .
